# Nonsurgical Management of the Neurogenic Bladder in Children

**DOI:** 10.1100/tsw.2008.146

**Published:** 2008-11-23

**Authors:** John Kryger

**Affiliations:** University of Wisconsin Hospital and Clinics, American Family Children's Hospital, Madison, WI, USA

**Keywords:** neurogenic bladder, spina bifida, intermittent catheterization, neurogenic bowel, urinary tract infections, pediatrics

## Abstract

The neurogenic bladder can have many serious consequences. However, with proper understanding of the disease spectrum, and with proper awareness of the surveillance and management tools, adverse outcomes can be minimized. The incidence of renal failure is uncommon. With proper medical therapy, one can achieve continence and minimize the risk of symptomatic urinary tract infections. There are a number of nonsurgical strategies described that can help in the management of the neurogenic bladder.

## INTRODUCTION

The neurogenic bladder can be a devastating condition. It causes a significant risk for urinary tract infections (UTIs), renal scarring, renal failure, vesicoureteral reflux, and incontinence. The management requires great responsibility and attentiveness by the patient, family, and physician. Neurogenic bladder arises from a multitude of conditions that include spinal dysraphisms, such as spina bifida, spinal cord injury, transverse myelitis, cerebral palsy, and others. These conditions come with a wide spectrum of severity and complexity. With spina bifida in particular, the level of the lesion does not always correlate to the exact nature of neurological deficiency.

Renal damage can begin within months of life among children born with a neurogenic bladder, such as in spina bifida. For example, the incidence of renal damage can approach 100% when there is poor bladder elasticity combined with detrusor-sphincter dyssynergia when it is not properly treated. However, with appropriate management, the incidence of renal failure is uncommon[1,2]. The goals are to achieve preservation of renal function, achieve continence, and achieve independence in daily care. These goals are often best achieved as part of a multidisciplinary care team involving pediatric urology, physical medicine and rehabilitation, neurosurgery, specialized nursing, and others.

## NORMAL AND NEUROGENIC BLADDER PHYSIOLOGY

In the normal bladder, there are two primary functions: urine storage and voiding. The autonomic innervation of the bladder is primarily mediated by the sympathetic nervous system arising from thoracolumbar nerves T10 through L2, the parasympathetic system arising from sacral nerves S2 through S4, and somatic innervation of striated external urethral sphincter mediated by the pudendal nerve arising from S2 through S4 (Onuf's nucleus)[3].

During storage, the normal bladder behaves as a highly compliant, elastic reservoir. It can accommodate volumes of urine appropriate to the size of the patient without significant increase in bladder pressure. The sympathetic nerves inhibit contraction of the detrusor muscle and stimulate smooth muscle tone at the bladder neck, in conjunction with pudendal nerve stimulation to enhance external urethral sphincter muscle tone to maintain strong resistance to urinary outflow, or leakage.

Voiding occurs when afferent nerves from the detrusor sense fullness and send signals to the central nervous system coordinated in the pontine micturition center. Efferent nerves send signals down the spinal cord, stimulating relaxation of the external sphincter and smooth muscle sphincter tone at the bladder neck by inhibiting sympathetic outflow along with synergistic detrusor contraction via increased parasympathetic activity. During voiding, the bladder contracts and urine is expelled with little outflow resistance, allowing complete evacuation of the bladder.

In the neurogenic bladder, various deficiencies arise in the storage phase, voiding phase, and detrusor-sphincter coordination. Bladder storage is compromised by multiple factors, including detrusor hyperreflexia in which the bladder experiences uninhibited contractions during storage that expel urine. Also, loss of bladder compliance and capacity can occur from fibrosis and collagen deposition amid the detrusor fibers, resulting in bladder wall thickening. In addition to the initial neurologic injury, worsening bladder compliance can be precipitated by exposure to prolonged elevated intravesical hydrostatic pressure[4]. Storage can also be compromised by an incompetent urinary sphincter, resulting in insufficient outlet resistance. A great contribution to neurogenic bladder management occurred when McGuire et al. reported a high incidence of renal compromise when bladder storage pressures exceed 40 cm of water pressure during the storage phase[5].

Voiding, or evacuation of the bladder, can be compromised by multiple factors. These include detrusor muscle hypotonia; loss of bladder compliance, resulting in a thick-walled bladder that does not empty effectively during voiding; and impaired relaxation of sphincter muscles arising from detrusor sphincter dyssynergia.

## EVALUATION

The evaluation of a child with a neurogenic bladder begins with a careful history of bladder and bowel habits. This includes questions to assess urinary frequency, urgency, intermittency, and incontinence. Other questions include any evidence of straining to urinate; any prevoiding mannerisms, such as freezing or curtsy mannerisms; and any history of UTIs. Incontinence needs to be characterized as urge-related episodes vs. stress-related episodes. It is also necessary to assess whether the bladder evacuates after each void or if the child is simply overflowing urine and never achieving complete bladder evacuation. For children on intermittent cath, it is important to know the frequency and timing of catheterization, the average urine volume, and whether there are any difficulties or obstacles to catheterization.

A careful history of bowel habits is equally important and must address the frequency of defecation and the character of stool, i.e., whether it is hard, soft, large, or small. Is there any history of pain or bleeding during defecation? Many parents will have an inaccurate history of their child's bowel habits. Some clues to constipation include bowel movements that are "unusually large for the child's size", "clogging of the toilet", pain or bleeding with defecation, and small, "rabbit pellet"–sized stool. A dietary history to assess the amount of dietary fiber, fluids, and caffeinated beverages is helpful.

The physical exam should include a careful abdominal exam, palpating for any masses, such as palpable stool or a palpable bladder. A careful genitourinary exam should confirm normal anatomy, as well as the presence of any skin redness or irritation that would suggest chronic urinary leakage. The back is carefully examined for any anomalies of the spine as well as any midline skin lesions, such as a hemangioma, nevus, isolated tuft of hair, or dimples. One should also note the presence and symmetry of the gluteal cleft. The anus is examined carefully for sphincter tone and any evidence of fissures, skin tags, or hemorrhoids.

The assessment continues with a radiologic evaluation and urodynamic study. The initial assessment must include a renal bladder ultrasound and assessment of postvoid residual, a VCUG and an urodynamic study that includes CMG/EMG, and pressure flow studies if spontaneous voiding can be initiated. Urodynamic studies can be enhanced by including video images in a video-urodynamic study. In the newborn child, urodynamic studies are often conducted at 6–12 weeks of age. One must consider that newborn urodynamics may harbor artifacts, such as CMG anomalies from agitation and motion artifact, or EMG anomalies in a male infant trying to void around a 7-french urodynamic catheter.

## TREATMENT

The nonsurgical management of the neurogenic bladder requires insights into the complex multifaceted elements that contribute to the neurogenic bladder behavior. Some children are able to demonstrate spontaneous voiding. If they are able to demonstrate safe urodynamics during storage, healthy appearing kidneys by ultrasound, and can eventually achieve continence and remain free of urinary infections, then this may be a suitable initial management plan. Incomplete bladder emptying and detrusor sphincter-dyssynergia are not absolute indications to begin bladder catheterization, but certainly are situations where a higher likelihood of risks can occur and very careful surveillance is warranted. Adjunctive measures to achieve continence often include medications, intermittent catheterization of the bladder, as well as surgical intervention for both bladder and bowel management. One must consider all options to try and achieve continence by school age.

### Medications

A variety of medications are useful in management of the neurogenic bladder. The most common medication utilized is anticholinergic therapy to suppress detrusor hyperreflexia. These medications treat detrusor hyperreflexia by blocking muscarinic cholinergic receptors. There are a multitude of agents available for use. Most commonly, oxybutynin is utilized with comparable efficacy and safety in tablet, liquid, and extended-release formulation[6,7]. Alternatives also exist, such as transdermal patches and intravesical instillation (tablets are dissolved in water and instilled directly into the bladder by catheterization). Agents that are available in a long-acting once-daily formulation may achieve better compliance with therapy, less side effects, and higher patient/family satisfaction. The newer agents may achieve better specificity to muscarinic, type 3 receptors and further minimize side effects. The most common side effects in children can involve facial flushing and dry mouth. However, there are numerous other anticholinergic side effects that may occur.

It is interesting, however, that in children with incomplete bladder emptying, cholinergic agonists, such as bethanecol, do not seem to achieve any improvement in bladder emptying. Furthermore, these tend to be complicated by rather significant side effects of diarrhea.

For children who void spontaneously, but experience urinary intermittency or incomplete bladder emptying related to detrusor-sphincter dyssynergia, one may try alpha-blocker therapy. Agents utilized in children include Flomax and Doxazosin. These have achieved success, are low risk to try, and can be followed for effectiveness by symptomatic improvement, as well as with a noninvasive uroflow study with postvoid residual[8].

For children with insufficient outlet resistance, alpha adrenergic agonists have been utilized. Sudafed can enhance smooth muscle sphincter tone at the bladder neck, which is well demonstrated by a common side effect of urinary retention reported in elderly patients from this medication. In children with a neurogenic bladder, it may be useful to try empirically with careful attention to the potential side effects.

### Intermittent Catheterization

In the 1970s, Lapides et al. published the first paper regarding safety and success of clean intermittent catheterization[9]. This is now a fundamental management technique in caring for children with neurogenic bladder. The neurogenic bladder is often compromised by decreased sensations of fullness, incomplete emptying, detrusor hyperreflexia, and detrusor-sphincter dyssynergia. By catheterizing the bladder, according to a timed schedule, one can achieve regular emptying, which can help to achieve continence, maintain low bladder pressures during filling, and ensure complete evacuation on a regular basis.

For many children, management of the bladder relies on a lifestyle of scheduled evacuation. A useful adjunct to a program of intermittent catheterization is a watch that is scheduled to alarm on a regular basis every 3–4 h to help remind the child to catheterize on a timed schedule and not based on their urge to urinate. We encourage the families to develop an awareness of the catheterized volumes, which can assist in interpretation of urodynamic studies and in troubleshooting episodes of urinary incontinence. When starting intermittent catheterization, it is very important to stress the use of a latex-free catheter and generous lubrication. Failure to lubricate adequately can result in increased risk of urethritis, painful catheterization, and mucosal prolapse. I favor a soft catheter in the male urethra to minimize the risk of urethral trauma and false passages.

By performing this in a simple, clean technique, one can minimize the complexity of the daily catheterized routine and allow for a much easier daily routine that achieves better compliance. Efforts to perform a sterile technique each time have not shown a significant difference in the incidence of UTI, although long-term randomized studies are limited[10,11].

Starting clean, intermittent catheterization will frequently result in chronic colonization of the bladder by bacteria. These bacteria are generally of low clinical consequence to urinary tract health if they are evacuated on a regular basis of at least four or more times per day. Children with chronically colonized bladders will occasionally experience a symptomatic UTI requiring a course of antibiotic therapy. However, we reserve use of antibiotics only for those infections associated with symptoms such as fever, urinary urgency, hematuria, or worsening incontinence. The urinary white blood cell count is an unreliable predictor for symptomatic urinary infections[12]. Cloudy or foul-smelling urine is a common phenomenon of the chronically colonized bladder, but does not necessitate antibiotic treatment. During such episodes, we encourage our patients to increase daily fluid intake, but avoid antibiotics that might disrupt the colonization of the bladder.

Overuse of antibiotics will contribute to formation of antibiotic-resistant organisms. The urine culture should be checked periodically for the presence of certain urease-positive organisms, such as *Pseudomonas* and *Proteus*. These are not suitable organisms to colonize the bladder and should be eradicated to minimize formation of urinary tract stones or biofilms[13]. In general, other bacteria, such as *E. coli* and *Klebsiella* sp., can safely colonize the bladder[14,15].

It is debated as to whether there is benefit to starting intermittent catheterization immediately in all children with neurogenic bladder vs. a delayed start based on urodynamic parameters, continence, or other symptoms. Some feel that all newborns should, in principle, be placed on CIC to ensure safe bladder pressures[16,17]. At our institution, we do not uniformly start all infants on intermittent catheterization, although we do teach the parents the technique of intermittent catheterization simply to encourage their familiarity with the technique and its possibility in the future of their child's health care, and also to help lessen any anxiety or aversion they may have to the technique. We reserve intermittent catheterization in infants to those who demonstrate incomplete bladder emptying or have poor bladder compliance during initial urodynamic studies.

## RECURRENT URINARY INFECTIONS

Recurrent UTIs are a common phenomenon among children with neurogenic bladder. This can occur in children who catheterize on a daily basis or those who urinate spontaneously with dysfunctional voiding.

In some circumstances, prophylaxis will help to decrease daily bacteria counts and can lower the incidence of symptomatic UTIs, especially when cycled with alternating antibiotics[18]. Oral prophylaxis is limited to agents with the lowest risk of long-term side effects, like nitrofurantoin or TMP/sulfa. However, the downside of daily prophylaxis is an increased likelihood of selecting antibiotic-resistant organisms[19,20,21,22]. The use of cranberry juice in this population does not appear to be effective in reducing colonization or UTIs[23]. Evidence-based guidelines for management of bacteriuria in children on intermittent catheterization are limited[24].

Another useful prophylactic agent is daily gentamicin bladder irrigation. This can be useful as a prophylactic agent as well as a treatment agent for possible antibiotic-resistant organisms. It is also usefully employed in combination with saline bladder irrigation to regularly clear the bladder of mucous in bladder augmentation patients. It can be performed one to three times per day. There is minimal absorption of gentamicin into the blood stream. There also appears to be a low risk for selecting gentamicin-resistant organisms. We utilize a solution of 480 mg gentamicin in 1 L of sterile water; 20 mL of this solution are instilled into the bladder after catheterization and left to dwell until the next catheterization[25,26]. The solution can be safely stored refrigerated.

## BOWEL MANAGEMENT

One cannot effectively manage a neurogenic bladder without careful attention to neurogenic bowel management. Constipation and stool retention often coexist with bladder dysfunction. The large volume of hard stool retained in the colon occupies space in the pelvis and places pressure on the bladder that compromises the ability to store and evacuate urine effectively. Many parents of children with a neurogenic bladder choose a technique of chronic constipation to achieve bowel continence. However, in some circumstances, this may compromise effective bladder management. It is useful to consider strategies of scheduled evacuation. This includes healthy dietary fiber intake and a daily stool softener, coupled with a method of daily evacuation to achieve effective bowel management.

Evacuation can be achieved with a daily rectal suppository, such as bisocodyl, glycerin, or some composite formulation like the Magic Bullet™ suppository[27]. Often, the suppositories are difficult to retain in the child with an incompetent sphincter tone. An effective technique of evacuation is a scheduled rectal enema. The cone adapter appliance allows it to fit snugly into the rectum with an incompetent sphincter such that an adequate volume of irrigant can be retained during filling. Once the cone is withdrawn from the rectum, there is usually a very adequate evacuation of stool from the distal colon. Certainly, the most effective means of evacuation are achieved by the Malone Antegrade Continent Enema (MACE) procedure, allowing for regular, complete evacuation of the colon[28,29].

## LONG-TERM CARE AND ASSESSMENT

The children with neurogenic bladder have a much higher incidence of learning disabilities. These are often not immediately recognized in social or clinic interaction. However, deficits in cognition and memory require very careful long-term surveillance of the child's daily routine of medications, catheterization, and bowel management[30]. Many families will try to initiate or achieve independence sooner than appropriate for their child, which often results in compromised compliance with the daily routines and subsequent compromised bladder and bowel management. Parents and caretakers must be carefully counseled to monitor activities of daily care, even when the child achieves some degree of independence. This is usually necessary until the age of 10 years and often beyond.

We also encourage behavior modification using techniques of positive reinforcement and repetition through scheduled daily routines. At each clinic visit, we carefully monitor blood pressure and obtain a urine sample for culture and sensitivity. Asymptomatic cultures will not require treatment, but are useful to document in case the family calls with symptoms of urinary infection, so that the best possible antibiotic is chosen to initiate therapy. It is also useful to ensure that the urine is clear of any *Proteus* or *Pseudomonas*.

Radiologic surveillance is suggested with an annual renal bladder ultrasound and urodynamic studies[2]. A VCUG to assess for occult reflux can be incorporated into a yearly video-urodynamic study. It seems that most urodynamic patterns and bladder behavior are well established by 16 years of age and there is lower risk of significant changes in adulthood. Some children are at low risk for compromise, such as those with an incompetent sphincter and continuous urinary leakage, and do not necessitate yearly urodynamics or VCUGs. Lab tests include yearly electrolytes, BUN, and creatinine in children with hydronephrosis, recurrent symptomatic infections, or high-grade reflux.

## CONCLUSION

Neurogenic bladder management has evolved greatly during the past 30 years. With proper management, the incidence of renal failure is rare. Furthermore, one can help children to achieve continence, independence, and self-esteem, and to minimize the risk of symptomatic UTIs. However, to achieve these goals requires insight into the underlying mechanisms of disease and awareness of the variety of resources available for the nonsurgical management of neurogenic bladder. With careful lifelong surveillance and judicious surgical interventions, these children can achieve great success.
